# A web-based intervention to support self-management of patients with type 2 diabetes mellitus: effect on self-efficacy, self-care and diabetes distress

**DOI:** 10.1186/s12911-014-0117-3

**Published:** 2014-12-14

**Authors:** Catherine H Yu, Janet A Parsons, Muhammad Mamdani, Gerald Lebovic, Susan Hall, David Newton, Baiju R Shah, Onil Bhattacharyya, Andreas Laupacis, Sharon E Straus

**Affiliations:** Li Ka Shing Knowledge Institute, St. Michael’s Hospital, Postal address: 30 Bond St, Toronto, ON M5B 1W8 Canada; Department of Medicine, University of Toronto, Toronto, ON Canada; Dhalla Lana School of Public Health, University of Toronto, Toronto, ON Canada; Applied Health Research Centre, St. Michael’s Hospital, Toronto, ON Canada; Department of Physical Therapy, University of Toronto, Toronto, ON Canada; Institute of Health Policy, Management and Evaluation, University of Toronto, Toronto, ON Canada; Institute for Clinical Evaluative Sciences, G1 06, 2075 Bayview Avenue, Toronto, ON M4N 3M5 Canada; Sunnybrook Research Institute, Sunnybrook Health Sciences Centre, Toronto, ON Canada

**Keywords:** Diabetes mellitus, Online systems, Patient self-management, Self-efficacy, Repeated measures modelling, Qualitative methods

## Abstract

**Background:**

Management of diabetes mellitus is complex and involves controlling multiple risk factors that may lead to complications. Given that patients provide most of their own diabetes care, patient self-management training is an important strategy for improving quality of care. Web-based interventions have the potential to bridge gaps in diabetes self-care and self-management. The objective of this study was to determine the effect of a web-based patient self-management intervention on psychological (self-efficacy, quality of life, self-care) and clinical (blood pressure, cholesterol, glycemic control, weight) outcomes.

**Methods:**

For this cohort study we used repeated-measures modelling and qualitative individual interviews. We invited patients with type 2 diabetes to use a self-management website and asked them to complete questionnaires assessing self-efficacy (primary outcome) every three weeks for nine months before and nine months after they received access to the website. We collected clinical outcomes at three-month intervals over the same period. We conducted in-depth interviews at study conclusion to explore acceptability, strengths and weaknesses, and mediators of use of the website. We analyzed the data using a qualitative descriptive approach and inductive thematic analysis.

**Results:**

Eighty-one participants (mean age 57.2 years, standard deviation 12) were included in the analysis. The self-efficacy score did not improve significantly more than expected after nine months (absolute change 0.12; 95% confidence interval −0.028, 0.263; p = 0.11), nor did clinical outcomes. Website usage was limited (average 0.7 logins/month). Analysis of the interviews (n = 21) revealed four themes: 1) mediators of website use; 2) patterns of website use, including role of the blog in driving site traffic; 3) feedback on website; and 4) potential mechanisms for website effect.

**Conclusions:**

A self-management website for patients with type 2 diabetes did not improve self-efficacy. Website use was limited. Although its perceived reliability, availability of a blog and emailed reminders drew people to the website, participants’ struggles with type 2 diabetes, competing priorities in their lives, and website accessibility were barriers to its use. Future interventions should aim to integrate the intervention seamlessly into the daily routine of end users such that it is not seen as yet another chore.

**Electronic supplementary material:**

The online version of this article (doi:10.1186/s12911-014-0117-3) contains supplementary material, which is available to authorized users.

## Background

Management of diabetes mellitus is complex, and involves controlling multiple risk factors that may lead to complications. However, care gaps exist: the Behavioral Risk Factor Surveillance System has estimated that only 68% of patients with type 1 or type 2 diabetes had HbA1c measured at least twice in the previous year [1], despite a recommendation from the American Diabetes Association that it be measured at least two to four times per year [2]. Given that patients provide most of their own diabetes care, patient self-management training is an important strategy for improving quality of care [3], particularly in the current era of patient-centred outcomes and comparative clinical effectiveness research [4]. Patient self-management interventions have demonstrated benefits in terms of both quality of life [5] and glycemic control [6], but participation is low [7], effectiveness wanes over time [6], and access to trained professionals to support self-management is limited [8]. Web-based self-management interventions are promising because they offer ease of access for patients who are computer-literate, and they can be scaled up with little cost [9]. Web-based media have improved patient knowledge, the extent of behaviour change, and clinical outcomes for a range of conditions [10]. However, principles of effective education, self-management support, and behaviour change have not been incorporated into current diabetes-related websites [11-13]. Reviews of existing diabetes websites showed that they presented didactic information of variable quality, they required advanced reading levels, and they followed a static, newspaper-format display, rather than harnessing the inherent advantages of websites, such as interactive technology, social support, and problem-solving assistance [11,13]. A systematic review of electronic diabetes-related tools found that they had moderate but inconsistent effects on a variety of psychological and clinical outcomes, including HbA1c and weight; tools that were more interactive tools were associated with continued website use and greater clinical improvement [10]. In addition, greater website use was correlated with greater clinical improvements: regular website users had greater reductions in HbA1c compared with intermittent users. Although this finding could be a consequence of the healthy user effect [14], addressing usability issues to increase the proportion of regular users may increase the effectiveness of interventions.

In a previous study, we developed an approach to address many of these limitations of existing web-based interventions [15]. In the current study, we tested the impact of this approach on self-efficacy, quality of life, self-care, blood pressure, cholesterol, glycemic control, and exercise promotion amongst people with type 2 diabetes.

## Methods

### Study overview

This study consisted of five phases: 1) development of the intervention, 2) feasibility testing, 3) usability testing; 4) refinement of the intervention, and 5) evaluation of the intervention using a cohort study and individual interviews. The study protocol and results of the first four phases are reported elsewhere [15,16]. We report here the results of Phase 5.

### Diabetes online companion: a web-based self-management intervention

The Diabetes Online Companion is a self-contained diabetes self-management website that was systematically developed according to self-efficacy theory. Self-efficacy refers to “beliefs in one’s capabilities to organize and execute the courses of action required to produce given attainments” [17]. Randomized controlled trials have shown that diabetes self-management education programs incorporating principles of self-efficacy are associated with improvements in knowledge [18], health behaviours [18,19], self-efficacy [18-20], HbA1c [18-21], weight [18], and microvascular complications [19].

Our intervention incorporated evidence-based content and behaviour-change strategies and followed the principles of user-centred design [15]. The website had four main components: 1) general information (static), 2) tailored information (interactive), 3) self-monitoring logs (interactive), and 4) a blog (interactive) (see Additional file 1 for sample screenshots). We posted a total of 53 blog posts over the intervention period, initially at a frequency of one per week. After four weeks of limited user activity, we increased the frequency of blog posts to two per week and added email prompts with each new posting. The topics, which covered medical content, diabetes-related news items, and practical issues, were selected on the basis of our feasibility and usability testing [15]. In addition, participants received weekly email reminders to visit the site or complete their self-management trackers, as well as notices of any new content [15].

### Cohort study

#### Participants

We conducted a single-arm pre-post cohort study. Consecutive series of individuals with diabetes were recruited from two family practice units and two endocrinology clinics in Toronto (one each from two academic health science centres). Those eligible for inclusion were aged ≥ 25 years with at least one of HbA1c > 7.0% (53 mmol/mol), systolic blood pressure > 130 mmHg, low-density-lipoprotein cholesterol (LDL-C) > 2.0 mmol/L, or body mass index (BMI) > 25 kg/m^2^. We excluded those who had Canadian Cardiovascular Society class 3 or 4 angina, did not speak English, were not available for follow-up, or had no regular access to the telephone and internet.

#### Outcomes

Website usage: We analyzed logs for the web server to assess the frequency and duration of specific components of the intervention [16]. Specifically, we collected data for the following variables: duration of use by individual users, frequency of use, site penetration, most frequently accessed tools and pages, and patterns of use over time.

Patient-centred outcomes: We assessed self-efficacy, our primary outcome, with the Modified Grossman Self-efficacy for Diabetes Scale, which has moderate to high reliability (Cronbach’s alpha = 0.51 to 0.86; Additional file 1) [22,23]. We selected self-efficacy because not only has it been validated in predicting and promoting patient behaviour change, but it also has been demonstrated to improve clinical outcomes [18,20,24,25]. We assessed self-care behaviour with the Summary of Diabetes Self-Care Activities Measure – Revised [26] and diabetes-specific quality of life with the Diabetes Distress Scale [27]. These patient-based outcomes were selected because they are relevant measures of knowledge use by patients.

Clinical outcomes: We collected data on HbA1c, systolic and diastolic blood pressure, LDL-C, and weight every three months. These outcomes were chosen to inform the sample size calculations in future trials.

#### Data collection

We obtained data for age, sex, ethnicity, education, self-reported health literacy, employment, duration of diabetes, complications, smoking status, medications, HbA1c, systolic blood pressure, LDL-C, weight, current use of and comfort with a computer and the internet, self-care score, self-efficacy score, and quality-of-life score at baseline. Outcome data were collected by means of patient-completed questionnaires. For the pre- and post-implementation phases, aggregates of patient-completed questionnaires were obtained every three weeks for nine months through web-based surveys, resulting in 12 data points for each phase. Health literacy was measured by a three-item validated questionnaire completed by the patients [28,29]. HbA1c and LDL-C were collected from medical records via chart audit. Systolic and diastolic blood pressures were measured by the research coordinator and were recorded as the average of three readings. Weight was also measured by the research coordinator. At the end of the study, each participant was asked to disclose whether he or she had used other web-based interventions and if so, whether those interventions employed text- or image-based didactic materials, interactive technology, or behavioural strategies. To assess for threats to validity from historical effects, we recorded secular events that might have affected our outcomes (such as diabetes-related news reports).

#### Sample size calculation

Using a range of correlations from 0.2 to 0.8, a significance level of 0.05, and a power of 80%, we calculated that a sample of at most 52 participants was required to detect a change of 0.5 units in self-efficacy score after the intervention (relative to the score before implementation). Differences of 0.1 to 0.5 in self-efficacy score have been correlated with metabolic control, eating behaviour, exercise behaviour, and other self-management behaviours [22,23]. A formula for paired mean comparisons was applied [30], and the longitudinal nature of the study increased its power [31]. A previous analysis reported a dropout rate of 20%–51% in studies of self-management [32]; we further adjusted the sample size to account for an expected dropout rate of 40%.

#### Data analysis

Linear mixed models were used to examine the effect of the intervention and time (intervention × time interaction) on self-efficacy, self-care, and diabetes distress. We selected these models to accommodate the complexities of typical longitudinal data sets for continuous outcomes; specifically, they allowed us to account properly for both within- and between-participant variability [33,34] and have been used in previous studies for similar analyses [35-37]. The models were also adjusted for age, sex, ethnicity, income (above or below Can $30, 000), education, employment, and health literacy, as each of these variables could affect the study outcomes [21,38,39]. The model examining the self-care outcome was also adjusted for interaction terms of the aforementioned variables with time. No additional interaction terms with time were included for other outcome models, because all additional interaction terms examined were non-significant. To avoid inflation of R^2^, all variables were specified a priori, and all interactions were tested simultaneously using a cut-off value of 0.30 [40]. Models were assessed by means of residual plots.

To assess the potential effect of missing income data for three of the participants, a sensitivity analysis (imputing income as both high and low) was performed. Missing health literacy data for 15 of the participants were imputed using the mode of the distribution, because 95% of the remaining participants were health literate.

Linear mixed models were also used to examine the effect of the intervention and time (intervention × time interaction) on secondary outcomes. These models were adjusted for age, self-efficacy score, income, ethnicity, and insulin use (for HbA1c and weight only). We also compared the effect of the intervention between users and non-users of the website. Finally, we used descriptive statistics to analyze website usage. R software version 2.1.15 was used for all analyses [41].

### Interviews

Individual interviews were conducted 2 to 21 weeks after completion of quantitative data collection. We used a purposive sampling strategy to recruit participants with a range of experiences and characteristics [42] (sex, age, ethnicity, duration of diabetes, educational attainment, income) from the broader pool of cohort study participants. We developed a semi-structured interview guide to elicit participants’ views regarding the following website features: acceptability, usability, strengths and weaknesses of the intervention, facilitators and barriers to its use, user satisfaction, and sustainability of use (Additional file 1). We made the website available during each interview, in case the interviewee wanted to show the interviewer something on the website.

All interviews were audiotaped and transcribed verbatim [43]. Transcripts were inductively analyzed to identify emergent categories and themes using a constant comparative approach [44]. Coding was conducted independently by three team members with expertise in qualitative research methods (CHY, JAP, SH) [44]. After coding an initial subset of interviews, a preliminary coding framework was developed on the basis of the emerging analysis, with discussion and consensus amongst the analysts [45]; the framework was then iteratively tested and refined with subsequent interviews [44]. Thematic saturation was attained with 21 interviews [42]. NVivo software (version 9) was used to assist with data management and retrieval. Techniques to ensure analytic rigour included use of multiple analysts, negative case analysis, and triangulation of the qualitative findings with the quantitative results [42,44,46]. Triangulation consisted of 1) examining the interview data through the lens of “effect on self-efficacy”, 2) corroborating qualitative findings with quantitative data, and 3) interrogating *how* the Diabetes Online Companion affected self-efficacy [46].

### Research ethics

The study was approved by the Research Ethics Boards of St. Michael’s Hospital (reference number 09–091) and Sunnybrook Health Sciences Centre (reference number 177–2009). All participants gave written and verbal informed consent.

## Results

### Cohort study

Of the 98 participants recruited, 81 had complete data collection for at least two time points (one before and one after the intervention was implemented) and were included in the analysis. The questionnaire response rate for these 81 participants was 83%. Patients’ characteristics and baseline self-efficacy, self-care, and diabetes distress are reported in Table 1 (Demographic characteristics and baseline values of observational cohort and qualitative study).

**Table 1:** **Demographic characteristics and baseline values of observational cohort and qualitative study**

		**Participants**	
		**Observational cohort (%, n = 81)**	**Qualitative study (%, n = 21)**
Sex	Male	44 (54%)	9 (43%)
	Female	37 (46%)	12 (57%)
Age (years)	20–39	7 (9%)	2 (10%)
	40–59	37 (46%)	7 (33%)
	60–79	36 (44%)	12 (57%)
	> 80	1 (1%)	0
Ethnicity	White	50 (62%)	17 (81%)
	Asian	24 (30%)	4 (19%)
	African American	6 (7%)	0
	Hispanic	1 (1%)	0
Duration of diabetes mellitus (years)	< 5	25 (31%)	6 (29%)
	5–9	16 (20%)	5 (24%)
	10–14	19 (23%)	3 (14%)
	15–20	16 (20%)	5 (24%)
	> 20 years	5 (6%)	2 (10%)
Education	< High school	1 (1%)	(0)
	High school	11 (14%)	1 (5%)
	College	21 (26%)	5 (24%)
	University	48 (59%)	15 (71%)
Employment status	Employed	45 (56%)	11 (52%)
	Retired	24 (30%)	7 (33%)
	Unemployed	7 (9%)	1 (5%)
	Disability	2 (2%)	(0)
	Student	3 (4%)	2 (10%)
Annual income (Can$)^†^	<15 000	17 (21%)	3 (14%)
	15 000 to 29 999	8 (10%)	4 (19%)
	30 000 to 59 999	22 (27%)	7 (33%)
	60 000 to 89 999	23 (28%)	6 (29%)
	>90 000	11 (14%)	1 (5%)
Insulin use	Yes	48 (59%)	7 (33%)
	No	33 (41%)	14 (67%)
Purpose of computer use*	Business	2 (2%)	1 (5%)
	Personal	24 (30%)	5 (24%)
	Both	54 (68%)	15 (71%)
Frequency of computer use*	< 1 time/week	3 (4%)	0%
	1–2 times/week	5 (6%)	0%
	3–6 times/week	9 (11%)	0%
	≥ 1 time/day	63 (79%)	21(100% )
Comfort with computer use	Somewhat uncomfortable	2 (2%)	0%
	Neutral	5 (6%)	0%
	Somewhat comfortable	26 (32%)	8 (38%)
	Very comfortable	46 (57%)	13 (62%)
	Did not respond	2 (2%)	0%
Frequency of Internet use for diabetes^‡^	< 1 time/week	66 (84%)	18 (85%)
	1–2 times/week	7 (9%)	1 (5%)
	3–6 times/week	5 (6%)	2 (10%)
	≥ 1 time/day	1 (1%)	0%
Comfort with Internet use	Somewhat uncomfortable	2 (2%)	0%
	Neutral	3 (4%)	0%
	Somewhat comfortable	29 (36%)	8 (38%)
	Very comfortable	47 (58%)	13 (62%)
	Did not respond	0%	0%
Self-efficacy; mean (SD)		4.61 (0.58)	5.11 (0.52)
Self-care; mean (SD)		3.35 (1.12)	3.31 (0.84)
Diabetes distress; mean (SD)		40.75 (16.21)	37.33 (14.74)
HbA1c; mean (SD)		7.64% (1.29)	7.17% (0.98)
		60.0 mmol/L (14.1)	54.9 mmol/L (10.7)
Systolic blood pressure (mm Hg); mean (SD)		129.24 (13.84)	124.10 (8.73)
Diastolic blood pressure (mm Hg); mean (SD)		76.03 (8.51)	74.71 (8.87)
LDL-C (mmol/L); mean (SD)		2.11 (0.78)	2.20 (0.73)
Weight (kg); mean (SD)		90.65 (21.76)	84.47 (15.13)

*Data missing for one participant.

^†^Based on Statistics Canada data for low income cut-off [43], we selected $30 000 as a minimal level of income comfortable for activities of daily living and self-management capability for our analysis.

^‡^Data missing for two participants.

*Abbreviation:*

*LDL-C* Low-density lipoprotein cholesterol.

### Website use

**Figure 1:**
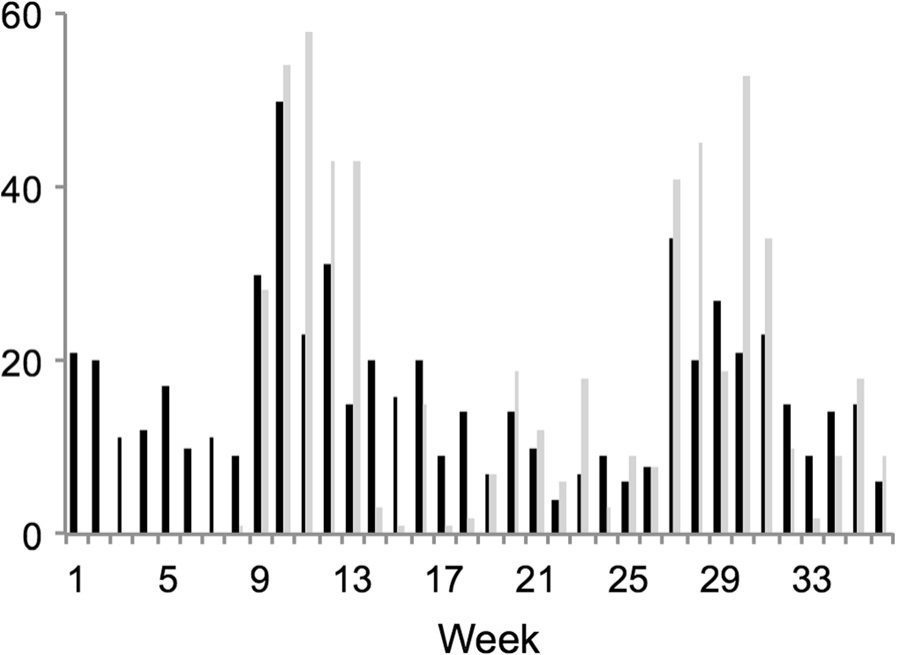
**Website login and blog use by week.** Black bar: Number of logins per week. Grey bar: Number of blog views per week.

The mean number of days on which users logged in during the study period was 8.2 days (standard deviation 13); the median was three days. The average frequency of use was 0.7 logins/month, or one visit every 5.8 weeks, distributed as follows: non-user: 11 participants (14%); infrequent user (<2 times/month): 61 participants (75%); frequent user (>2 times/month): seven participants (9%); heavy user (>1 time/week): two participants (2%). Website usage across all users ranged from 4 to 50 logins/week (median 14.5/week), with peaks of 50 logins in week 10 and 37 logins in week 27. Increased use of the website during those weeks appeared to be driven by the blog. In general, website use appeared to parallel blog use, with users visiting the blog repeatedly during the same login or visit (Figure 1). The most-accessed pages during week 10 were the blog (regarding medication log, supplements, and insulin) (34% of hits) and the blood pressure (8%) and medication (9%) logs. For week 27, the most-accessed pages were the blog (regarding foot care) (44% of hits), “My blood glucose log” (32%), and “7 steps to take care of your feet” (3%).

Overall, the most frequently accessed tools, for both first-time and return users, were the blog, followed by “My blood glucose log”, “My medication log”, and “My activity log”. Regarding site penetration, users viewed 6.6 pages per session, spending an average of 5 minutes 43 seconds on the site, and 1 minute 39 seconds per page.

### Blog use

Within the blog section of the website, there were a total of 569 page views by 35 participants over the study period, with peaks at week 10 (54 views), week 27 (43 views), and week 30 (53 views), corresponding to blog entries about the medication log, supplements and insulin, and foot and kidney care, respectively. A total of 13 comments responding to the blog postings were submitted by five participants. These comments took the following forms: 1) responding to the blog (agreement or disagreement); 2) requesting help with or providing feedback on the website; 3) requesting help with self-management; 4) offering assistance, empowerment, and their own solutions (including food recipes); 5) self-reporting behaviour change; 6) sharing responses to medication; and 7) warning others about interactions with health care providers.

### Use of interactive and static tools

Overall, 47 (67%), 63 (90%), and 43 (60%) of 70 users visited static, interactive, and log pages, respectively, at least once. These users had a mean of 3.4, 4.5, and 9.3 visits/user to each of these page types, respectively.

### Patient-centred outcomes

**Figure 2:**
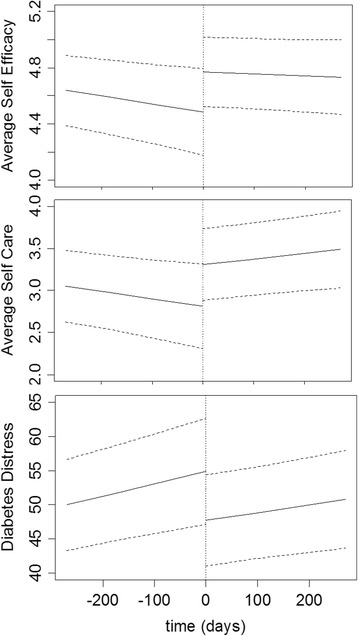
**Self-efficacy, self-care, and diabetes distress nine months before and nine months after intervention implementation.** Reference categories used in the plot were as follows: female, mean age 57.34 years, employed, university education, income > Can$30,000, adequate health literacy, white.

Self-efficacy: Despite a significant short-term increase in self-efficacy score immediately after implementation of the intervention (0.13; 95% confidence interval [CI]: 0.06, 0.20; p < 0.0004), by nine months, this outcome had not increased significantly more than expected from its pre-implementation trajectory (effect: 0.12; 95% CI: −−0.028, 0.263; p = 0.11; Figure 2 and Table 2).

**Table 2:** **Summary statistics of psychological and clinical outcomes (with 95% confidence interval), at implementation of intervention and 9 months later**

**Outcome**	**Immediate change (at time of intervention)**	**p-value**	**Change in outcome at study end, over expected value**	**p-value**
**Self-efficacy**	0.13 (0.06, 0.20)	0.0004	0.12 (−0.028, 0.263)	0.11
**Self-care**	0.26 (−0.17, 0.68)	0.24	0.44 (0.23, 0.63)	<0.0001
**Diabetes distress**	−2.29 (−3.76, −0.81)	0.002	−1.84 (−4.81, 1.12)	0.22
**HbA1c**	0.37 (−0.11, 0.85)	0.13	−0.055 (−1.13, 1.02)	0.93
**Weight (kg)**	0.37 (−3.00, 3.74)	0.83	−1.10 (−9.31, 7.12)	0.77
**Blood pressure (mm Hg)**				
**Systolic**	1.79 (−5.87, 9.26)	0.66	−5.89 (−18.26,12.37)	0.53
**Diastolic**	0.43 (−4.13, 4.98)	0.85	−8.22 (−19.18, 2.74)	0.14
**LDL-C (mmol/L)**	−0.0006 (−0.26, 0.26)	0.996	0.14 (−0.55, 0.82)	0.72

*Abbreviation:*

*LDL-C* Low-density lipoprotein cholesterol.

Self-care: The self-care score improved by 0.44 (95% CI: 0.23, 0.63; p < 0.0001) beyond what was expected at nine months (Figure 2 and Table 2).

Diabetes distress: Despite an immediate short-term decrease in diabetes distress score (−−2.29; 95% CI: −3.76, −0.81; p = 0.002), by nine months, this outcome had not decreased significantly over what was expected (effect: 1.84; 95% CI: −4.81, 1.12; p = 0.22; Figure 2 and Table 2).

There was no interaction with insulin use by time or intervention for any of these outcomes.

Self-care scores were positively correlated with age (0.04/year, 95% CI: 0.02, 0.06), p <0.001).

Diabetes distress varied with age and sex: younger female participants had greater diabetes distress.

When we conducted the sensitivity analysis with missing values for income assumed to be below $15,000 and missing values for health literacy assumed to be the mode, there were no changes in results for self-efficacy, self care, or diabetes distress.

### Clinical outcomes

Seventy-three of the participants were included in the analysis of clinical outcomes. The other eight participants were excluded because of missing data for HbA1c, blood pressure, LDL-C, or weight within 90 days of the self-efficacy data or because no data were obtained after implementation of the intervention. The intervention had no effect on HbA1c, blood pressure, LDL-C, or weight in either the unadjusted or the adjusted models (Table 2).

Comparison of users and non-users:

A total of 70 participants (86%) used the website at least once, whereas 11 (14%) did not use the site at all. At the nine-month follow-up after implementation of the intervention, there was no difference between users and non-users in terms of self-efficacy (0.15 vs. 0.13, p = 0.35) or self-care (0.18 vs. 0.13, p = 0.21). Users had a greater reduction in diabetes distress than did non-users (−4.7 vs. -0.9, p < 0.0001). There was no difference in the effect of using the intervention on any secondary outcomes, with the exception of diastolic blood pressure (users: +3.27 mm Hg; non-users: −1.6 mm Hg; p = 0.014; Additional file 1).

### Interviews

Twenty-one individuals (Table 1) participated in an interview. The sample consisted of White and Asian men and women of various ages, duration of diabetes, educational attainment, and employment status, who used computers frequently and were comfortable with using the internet. Analysis of the interviews revealed numerous themes, four of which were most relevant to interpretation of the cohort study’s negative results, in particular, exploration of why participants used the website to only a limited extent. Additional themes will be the focus of future publications. The following four themes are considered here: 1) barriers and facilitators of website use; 2) patterns of website use, including the role of the blog in driving site traffic; 3) general feedback on website characteristics; and 4) potential mechanisms for the effect of the website on self-efficacy, behaviour change, and diabetes distress. Representative quotes for each theme appear in Table 3.

**Table 3:** **Themes identified and representative quotes from in-depth interviews**

**Theme/subtheme**	**Representative quotes**
**1) Barriers and facilitators of website use:**	
Barriers to website use	
Patient-related barriers	
a) Competing health concerns	“Being 4 cancers in my life”. [3B53, 78-year old woman]
	“it’s not just diabetes that I deal with”. [2B16, 37-year old woman]
b) Competing life concerns	“I think for me, health issues surround migraines and fatigue. By the time I was home and had any free time, there wasn’t a lot there. And then life style and other stresses the business and other stresses the business…” [2B16, 37-year old woman]
c) Lack of motivation	“It’s a me thing, as opposed to a site thing”. [2B09, 47-year old woman]
	“Laziness”. [3B52, 65-year old woman]
d) Frustration with diabetes	“Part of it is, when you see the blood sugar is really high, I already know it’s high. I’m not taking the medication. So to log the fact that they are high, ends up making you more frustrated. So why do that”. [2B09, 47-year old woman]
e) Futility regarding diabetes	Sometimes I think no matter what I do it’s not going to matter because you read about this… they all end up on dialysis… they all, you know…” [2B09, 47-year old woman]
Website-related barriers	
f) Login and password requirement	“I keep forgetting my password”. [3B27, 58-year old woman]
	“The ability to change the password is good as well… Because I think that helps in returning, otherwise I would always go back and find your original email”. [2B16, 37-year old woman]
g) Limited computer and/or internet access	“I didn’t have too much time to look at it ’cause I don’t have a computer at home [3B39, 67-year old man]
	“I wish I can have a mobile like app”. [3A10, 52-year old woman]
	“on the road for work and limited web access”. [1A13, 47-year old man]
h) Onerous nature of data entry to use logs	“Um, but cause I did go in and I did try and do the tracking and I think cause I thought that was on an ongoing basis was the most useful part of it. But it was kind of a pain in the neck to use it… and kind of a pain in the ass getting where I wanted to go. I put some information and I wanted to delete it and I don’t know if I ever succeeded in getting rid of it”. [2B01, 48-year old man]
Facilitators of website use	
i) Perceived reliability of information	“It has to be really, really tuned in or connected to the latest developments either at [hospital name] or the world… With tons of information by real authorities. If I had a diabetic questions, I thought you guys might answer it, I would consider this to be an authoritative source”. [2B01, 48-year old man]
j) Reminders, prompts of new content	“Actually love getting the reminders, I really do. In this day in age everybody is busy, but I’m really good about it. The day I get the reminder, I find time to go in, I read the blogs… As I said, if you told me there was something new under a certain heading, I would definitely go in. I just like the reminders, in this fast paced world you don’t always have time for things and the last thingyou think about is yourself. So I think the reminders are a great thing, and I always look”. [3B09, 61-year old woman]
k) Increasing comfort with internet use	“Slowly I got used to [the internet] and since that time I utilize the possibility to look after whatever is interesting to me”. [3B53, 78-year old woman]
	“I starting writing a lot of emails to lawyers and the public trustee so I’m kind of used to no, more so than before, tracking stuff online. Kind of going paperless is neat”. [2B01, 48-year old man]
**2) Patterns of use of the website, including role of blog in driving site traffic**	
a) Goal-directed use	“And that’s kind of how I use the tool. It’s not as much a “Do I go in daily and read a new section each day?” No, it’s when I have a specific concern that I hone in on it to a degree”. [2B09, 47-year old woman]
	“Right, so there are a lot of places [in the website to which] you can go. But if those places don’t impact you… For example, if you don’t have high blood pressure, if it doesn’t impact you, you are less likely to go back to it. Then when it impacts you, then you know the resource is there”. [2B09, 47-year old woman]
b) Use in context of current concern	“Because the bottom of my toes is not sensitive and the side of my foot is not sensitive. So I looked up this website and tried to find out what could I do and is there any kind of help that I could find”. [3B52, 65-year old woman]
-Motivation for repeated use	“The foot complication is the one I usually look up”. [3B52, 65-year old woman]
-To gage urgency	“I would look something to see if I thought if it was urgent”. [3B13, 54-year old woman]
	“Now, I have to say yesterday in desperation I actually tried to look up some medication information on this website…” [2B09, 47-year old woman]
c) Role of blog in driving site traffic:	“When new blogs coming up then I read it. Not always at once, but still eventually”. [3B53, 78-year old woman]
Dual roles of blog	“‘Ask the Expert’ should remain open all the time… And maybe it’s not just ‘Ask the Expert’; maybe it’s ‘Ask someone’. So you need an ‘Ask the Expert’ section and ‘Ask the Fellow Patient’ section”. [2B09, 47-year old woman]
i) Opportunity for anonymous request for information	“It provides an opportunity where people however anonymous they want to be and it might be something someone could be shy asking a heath care practitioner meanwhile go ahead and ask on something like that. So in that sense, I think that’s useful”. [2B16, 37-year old woman]
ii) Fostering a sense of community	“It’s interesting because people will come with a question through [the blog, such as] “Well, this worked for me”, or “This will work for everyone.” It is a sort of communal sense and conversation”. [3B53, 78-year old woman]
	“So, I think learning to develop your support systems is extremely important for a diabetic. And that having a forum where even if you don’t have a lot of people in your life that you can talk to about this, but having a forum where maybe you can go on and have an online community can be very helpful. Provided that they understand that any information that they read has to be verified”. [3B13, 58 year-old woman]
-Social support	“For example, if you are feeling yourself alone and loneliness is not a very good thing health-wise. It leads to depression and everything else and if you feel that you have to communicate, and then communicate here”. [3B49, 46-year old man]
-Though this was not a universal sentiment: others felt a lack of connection	“Sometimes with a blog it can be communal and it can be conversation, but it can also make people feel disconnected because: how comfortable are they in conversation?” [2B16, 37-year old woman]
iii) However, tension between desire for online community and fear of ridicule, timidity	“Because we are afraid that we are putting a stupid question”. [3B53, 78-year old woman]
	“I found at times, responding to other posts, that [I felt] shy about responding. But I did go ahead and do that even though I thought at times that’s not necessarily my usual style”. [2B16 37-year old woman]
	“It might take time to catch on, because people who are feeling shy about it may need to see what other people do with it for quite a while”. [2B16, 37-year old woman]
	“I think some people are shy about how they write.” [2B16, 37-year old woman]
	“I’ve just never been interactive…” [3B09, 61-year old woman]
iv) Other reasons for not contributing to the blog:	
-Nothing new to contribute	“But I’m not a poster. I read, I learn, I just don’t put my 2 cents in. [I don’t post because] I would say the same thing – why would I bother?” [3B09, 61-year old woman]
-Reliability of content: Balance between evidence anecdote, trusted source versus patient centred	NO “I find for the most part it’s the blind leading the blind. I guess this one is being moderated but by and large you have a bunch of people who don’t know anything kind of spewing forth”. [3B13, 54-year old woman]
	YES “However, the way this one is designed essentially it goes and it’s approved or reviewed before being posted in the first place. Which I think is a good style”. [2B16, 37-year old woman]
-Perceived relevance to specific demographic population	“I don’t know what the ages are but I’m thinking a younger person would be more familiar with communication in a way that is not face to face, more so”. [2B16, 37-year old woman]
**3) General feedback on website characteristics:**	
a) Accurate and comprehensive	In fact, what I noticed is that their seemed to be more than what I would have anticipated… [2B01, 48-year old man]
	So there are lots of things on the website that really did help. So things like how to control the blood pressure and how to do all those other… there is lots of things that help. [2B09, 47-year old woman]
b) Easy to navigate	“The website itself is easy to navigate. And I think that list is very good and I’ve found that every time I have looked, [the answer] could typically could fall into one of those categories. The answer might not be there, but I know where to start to look”. [2B09, 47-year old woman]
c) But could be more relevant:	
-Want practical solutions	Maybe too many people are inundated with expert answers and not enough with real life answers. [3B09, 61-year old woman]
	But that when you need it the most, that’s when I found it didn’t have everything I needed. It was still missing… the next piece; the next piece is what happens when you are in that mess? [3B49, 46-year old man]
	Yeah, it combines that very practical app, ok so here is an endocrinologist talking about blah, blah, blah, new scientific in road into diabetes… ok, that’s great on an intellectual level… What does this mean on a practical level? For those that want to and can handle the scientific information, perfect. And those of us who can’t or don’t want to then there’s other things on the site. [3B13, 54-year old female]
	What do you value about each type of information, the practical versus medical evidence based? 70 or 80 [percent of it practical] versus 30 and 20 for the medical. [3B53, 78-year old woman]
	But nowhere does it tell you how to deal with it. So, what I’m looking for and what I was looking for in that peer support was other people in the same position who have found solutions to issues that typical websites don’t tell you [2B09, 47-year old woman]
**4) Potential mechanism of impact of website on self-efficacy, behavior change and diabetes distress**	
a) Unanticipated pattern of use of DOC	“I actually I haven’t done it lately but when I first got this I searched a lot of stuff, I was interested so I did search. I printed it out I actually have some in a binder, which I have referred to on occasion. It’s nice to have something black and white that you can refer to. That’s why I like certain websites, because you can print it off and read it. I can search other references and I’ve done that. And I keep a binder and I refer to it when…” [3B09, 61-year old woman]
-Print out	
b) Unanticipated impact of reminder emails	“There were the reminders that you hadn’t visited the site in a while. [When I got those reminders], I said, I will answer it later…
	This companion is a good reminder, I heard about it before but then let’s go back and check to make sure that I’m understanding properly…” [3B27, 58-year old woman]
	“Constantly reminding me about the things that we need to be aware of. Most of us know but the Online Companion was a good reminder and got me thinking of things that I need to constantly do (some that we conveniently forget)”. [3B55, 58-year old woman]
c) Unanticipated role of DOC in “kickstarting” self-management behaviors	“Because of the recording of activity… I started… it guilted me into starting to record my activity and once I started recording it because also coming off of the Metformin, I started recording it there and then I switched to recording it in books and now I simply record it on the calendar”. [1A06, 62-year old woman]

*Abbreviations:*

*St. Mike’s* St. Michael’s Hospital, Toronto, ON, Canada.

*App* application.

*DOC* Diabetes Online Companion.

1) Barriers and facilitators to use: Participants stated that they struggled with competing health and life concerns. They reported that it was “not just diabetes” that they dealt with (Table 3; 1a) and that they had to manage other concurrent medical conditions (Table 3; 1b). They spoke about their attempts to balance *illness work* with *everyday life work*; they found that after completing the latter, “there wasn’t a lot” of time or energy left for self-management of their disease, much less to use the website (Table 3; 1b). Some participants identified lack of motivation as “a me thing, as opposed to a site thing”, while others commented that “laziness” (Table 3; 1c) was a barrier to use.

Participants’ attitudes toward diabetes also coloured their approach to self-management and thus their use of the site. In particular, participants reported feeling frustrated with the uncontrolled nature of their disease, and the collection of self-monitoring information that showed a lack of metabolic control exacerbated this frustration (Table 3; 1d). Similarly, some participants said that they were sometimes overcome with a sense of futility. They perceived that regardless of their actions, some outcomes such as dialysis were inevitable (Table 3; 1e); hence, they saw no value in learning about the disease or in trying to self-manage the disease or use the website.

Eleven (52%) of the 21 interview participants said the requirement for a login and password prevented them from using the website because they often forgot their password (Table 3; 1f). Others were limited by poor computer or internet access and said they would prefer a mobile solution (Table 3; 1g). Finally, some participants noted that the onerous process for correcting error in log entries discouraged them from using the self-management tools (Table 3; 1h).

In contrast, other website characteristics appeared to encourage users to visit and return. The perceived reliability of the website’s information and the perception that it was an “authoritative source” drew users (Table 3; 1i). In addition, email reminders prompted them to return; such prompts seemed well-suited to what users characterized as a “fast-paced world” and served as effective reminders to make diabetes self-care a high priority (Table 3; 1j). Similarly, routinization of the online experience appeared to routinize use of the internet for certain aspects of health care. For example, participants reported that increasing their use of the internet and expanding their scope of internet activities created a new norm for internet usage, such that it became more commonplace for them to “look up whatever is interesting to me”, “track stuff online”, and embrace “going paperless” (Table 3; 1 k).

2) Patterns of website use, including role of the blog: Participants said that use of the website was driven by their individual context and circumstances. Rather than browsing at random, users said they were goal-directed: when they had a specific concern, they focused on that area of the website (Table 3; 2a). For example, one participant was initially motivated to visit (and subsequently continued to visit) the foot care section of the website because of her foot symptoms (Table 3; 2b). Participants also commented that they used the website to gauge the urgency of their concerns and to try to obtain immediate answers to their questions (Table 3; 2b).

We explored potential reasons for the unexpected finding that the blog was the most frequently accessed tool and appeared to drive website usage. Participants identified dual roles of the blog in providing a forum for both “expert” and “fellow patient” advice (Table 3; 2c). “Fellow patient” advice consisted of submitting suggested content or commenting on blog posts (i.e. participating in a discussion thread). For patients who were otherwise uncomfortable with asking questions of a health care provider, the blog afforded them the opportunity to obtain “expert” medical answers anonymously (Table 3; 2c,i). The provision of “fellow patient” advice was characterized as promoting a sense of community that some participants felt might combat their sense of isolation (Table 3; 2c,ii). However, this was not a universal sentiment, and some participants felt uncomfortable with and disconnected from the blog (Table 3; 2c,ii). In addition, there was a tension between a desire for online community and a fear of “looking foolish”. For example, some participants said they were afraid of “putting [up] a stupid question” or surmised that others were “shy about how they write” (Table 3; 2c,iii). Other reasons offered for not contributing to the blog included participants’ perceptions that they had nothing to offer, that the blog was not their preferred mode of communication, and that they preferred face-to-face communication (Table 3; 2c,iv). There were mixed views regarding the reliability of the blog content: one user commented that a blog represented “the blind leading the blind”, but others said they were reassured by the fact it was moderated by an expert (Table 3; 2c,iv).

3) General feedback on website characteristics: Participants commented on their impressions of the website overall and provided feedback concerning general features that were appreciated. Participants perceived that the website was accurate, comprehensive (Table 3; 3a), and easy to navigate (Table 3; 3b). While they appreciated the website’s provision of “evidence-based medical content”, they reported a desire for more practical solutions and “real life answers” (Table 3; 3c) and spoke about finding a balance between these two characteristics.

4) Mechanisms of effect of the website on self-efficacy, behaviour change, and diabetes distress: Despite apparently limited use of the website, the intervention appeared to have an effect on self-efficacy, behaviour change, and diabetes distress. Deeper exploration of the data regarding patterns of use and website features uncovered factors that might account for these quantitative findings. For example, rather than returning to the site to revisit and review items, some participants reported that they printed items of interest from the website and subsequently referred to these paper copies (Table 3; 4a). The use of reminder emails also had an effect. Participants reported that these emails not only prompted them to return to and log into the website, but also encouraged them in their own self-management (Table 3; 4b). Finally, one participant noted that the website had kick-started her self-management behaviours, by initially “guilting” her into recording them online. Although she did not subsequently login to the website to record these behaviours, she did continue to record them on paper.

## Discussion

We found that a self-management website for patients with type 2 diabetes led to no improvement in self-efficacy, diabetes distress, or clinical outcomes over the study period. However, there was an improvement in self-care (a secondary outcome), and the group that used the website experienced significantly lower diabetes distress than those who did not use it. Despite a user-centred design process and an increase in the frequency of blog posting from weekly to twice weekly, use of the website (as ascertained by login records) was limited. Our interviews revealed that both patient-related factors (e.g. competing health and life concerns, a sense of futility) and website-related factors (e.g. requirement for login, limited computer or internet access) limited use of the website. We also found that participants were motivated to access the website on the basis of their current needs and concerns, as well as new blog postings, with the blog fulfilling a need for both “expert” medical content and peer support and a sense of community.

These qualitative findings have confirmed the importance of website features such as the reliability and authoritativeness of information [47], as well as the use of blogs [15] and reminders [48] for continued engagement of users. Our findings also emphasize the need to provide a greater proportion of “practical” patient-centred content. A recent qualitative study analyzing 3005 diabetes-related blog posts showed that the most influential blogs were those written by patients and that only 10% of blogs cited biomedical literature [49], highlighting the dual needs for reliable, evidence-based content and engaging patient-based content.

Our data also suggest that mobile devices are a potential avenue through which to improve accessibility and use of a self-management site. A Cochrane review of computer-based diabetes self-management interventions identified 16 randomized controlled trials, which showed a small effect on glycemic control (−2.3 mmol/mol or −0.2%, p = 0.009), with the mobile phone subgroup experiencing a greater effect (−5.5 mmol/mol or −0.5%, p < 0.00001) [50]. Given the increasing preference for mobile devices over desktop computers [51] for health information resources [52], mobile technology may overcome the barriers to website access and use that we identified, through greater integration with patients’ existing routine, such that self-management is no longer seen as additional “illness work”. However, as with web-based technology, a systematic approach to development, testing, implementation, and evaluation of mobile health technology is warranted. Although such technologies are proliferating, with over 736 applications related to diabetes alone, their usability and clinical effectiveness are variable [53], and concerns exist regarding their effectiveness and safety, as well as the security of personal health information [54]. Our findings regarding user engagement with web-based technology echo those for mobile technology: an evaluation of 10 mobile diabetes applications emphasized the importance of user-centred design, an engaging interface, and context-driven use [55].

Competing health concerns were identified as a barrier to web-based self-management. Patients’ adherence to diabetes care is affected by multimorbidity (e.g. depression), which in turn directly affects self-management ability and competes for time and attention [56]. For example, patients with a greater number of comorbidities placed a lower priority on diabetes and had worse diabetes self-management ability [57]. Future interventions should consider strategies, such as shared decision-making and priority-setting, to empower patients with multiple comorbidities to optimize their self-care [58]. For example, a patient may identify mood management as a priority, which is key to subsequent self-care. Thus, greater integration with the person’s cognitive, emotional, and health information-seeking preferences, daily living routine, and health context through the use of patient-based content, mobile devices, and individualized decision-making, may be further strategies to maximize website use and reduce intervention attrition.

Finally, our results may be extrapolated to other chronic diseases. In particular, our finding of the need for tailored content and peer support, balanced with concerns regarding information reliability and confidentiality, is applicable to other strategies for managing chronic disease. For example, a systematic review of the benefits and limitations of social media in the context of chronic disease identified benefits (increased interaction and social support, tailored and accessible information) and limitations (quality concerns and lack of reliability, confidentiality, and privacy) [59] to those we identified. Similarly, our finding of a reduction in diabetes distress in conjunction with no improvements in clinical outcomes echoes findings from intervention strategies targeting other chronic diseases. For example, another systematic review examining the effect of social media on psychological and physical outcomes in chronic disease found a relatively large body of evidence demonstrating psychological benefit (19 identified studies) but limited evidence for physical outcomes (4 identified studies) [60].

This study was limited by its non-randomized design. However, we employed a repeated-measures design that permitted reliable assessment of baseline self-efficacy. Although our primary outcome (self-efficacy) was a non-clinical outcome, it is a validated predictor of patient behaviour change and clinical outcomes [18,20,24,25]. The infrequency of website use likely limited the effect of this intervention, but we obtained valuable insights regarding mediators of website use through our individual interviews. The qualitative evaluation was conducted by individuals who were also involved in developing the intervention, which created a potential for bias; however, we guarded against this bias by including individuals who were not involved in designing the website as members of the qualitative analytic team and by having three coders. As such, we were able to obtain and report critical feedback that participants openly shared. Study strengths include the use of multiple repeated measures, the use of validated outcomes, dual coding of all transcripts, and triangulation of the qualitative findings with the quantitative results [42,44,46].

## Conclusion

Increasing use of the World Wide Web by consumers for health information and ongoing revolutions in social media are strong indicators that consumers are welcoming and demanding a new era of technology in health care. However, full potential of this technology is hindered by limited uptake and high attrition rates. Use of the Diabetes Online Companion may be optimized by integrating a mobile interface, emphasizing “practical” patient-centred content such as a patient-led blog, and including a “prioritization” feature to help users with competing concerns. Our research findings have shed light on these limitations by identifying characteristics associated with website use and attrition and suggesting strategies to reduce website attrition as a way to potentially optimize clinical outcomes.

## Additional file

Additional file 1:
**Appendix A: Representative Screenshots of the Diabetes Online Companion. Appendix B: Description of primary outcome scales. Appendix C: Semistructured interview guide. Appendix D: Effect of Intervention comparing users and non-users (after adjustment for time). Appendix E: References.**
