# Supplementary material for: A web-based intervention to support self-management of patients with type 2 diabetes mellitus: effect on self-efficacy, self-care and diabetes distress
Source: BMC Med Inform Decis Mak. 2014 Dec 14;14:117. doi: 10.1186/s12911-014-0117-3 (PMC4272538; doi:10.1186/s12911-014-0117-3)
Supplement: Additional file 1: — Appendix A: Representative Screenshots of the Diabetes Online Companion. Appendix B: Description of primary outcome scales. Appendix C: Semistructured interview guide. Appendix D: Effect of Intervention comparing users and non-users (after adjustment for time). Appendix E: References. [file 12911_2014_117_MOESM1_ESM.pdf]

Appendix A: Representative Screenshots of the Diabetes Online Companion

a) Screenshot of Sample Topic Page "Blood sugar":

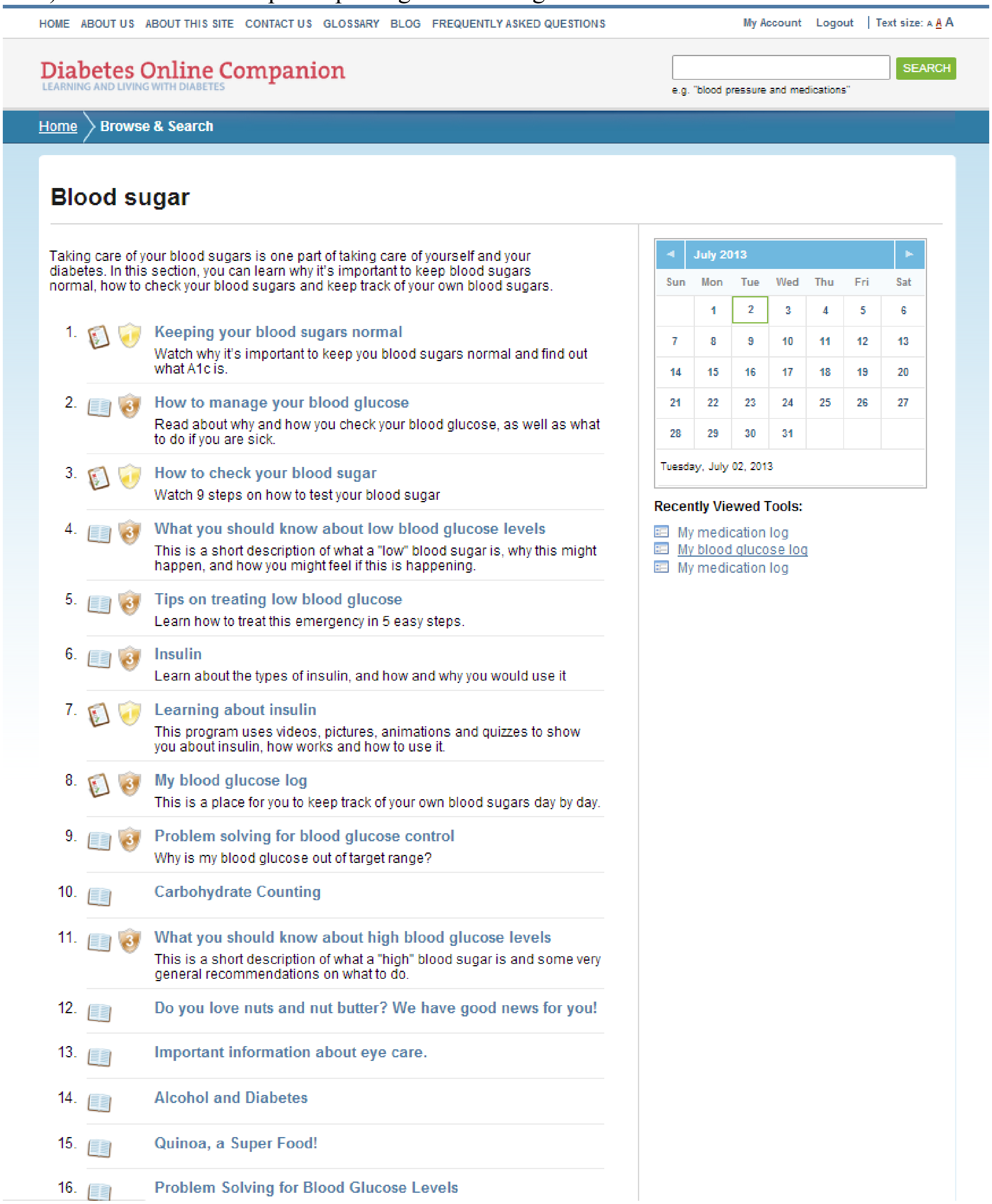

HOME ABOUT US ABOUT THIS SITE CONTACT US GLOSSARY BLOG FREQUENTLY ASKED QUESTIONS My Account Logout | Text size: A A A

Diabetes Online Companion
LEARNING AND LIVING WITH DIABETES

SEARCH
e.g. "blood pressure and medications"

Home > Browse & Search

Blood sugar

Taking care of your blood sugars is one part of taking care of yourself and your diabetes. In this section, you can learn why it's important to keep blood sugars normal, how to check your blood sugars and keep track of your own blood sugars.

1. **Keeping your blood sugars normal**
   Watch why it's important to keep you blood sugars normal and find out what A1c is.
2. **How to manage your blood glucose**
   Read about why and how you check your blood glucose, as well as what to do if you are sick.
3. **How to check your blood sugar**
   Watch 9 steps on how to test your blood sugar
4. **What you should know about low blood glucose levels**
   This is a short description of what a "low" blood sugar is, why this might happen, and how you might feel if this is happening.
5. **Tips on treating low blood glucose**
   Learn how to treat this emergency in 5 easy steps.
6. **Insulin**
   Learn about the types of insulin, and how and why you would use it
7. **Learning about insulin**
   This program uses videos, pictures, animations and quizzes to show you about insulin, how works and how to use it.
8. **My blood glucose log**
   This is a place for you to keep track of your own blood sugars day by day.
9. **Problem solving for blood glucose control**
   Why is my blood glucose out of target range?
10. **Carbohydrate Counting**
11. **What you should know about high blood glucose levels**
    This is a short description of what a "high" blood sugar is and some very general recommendations on what to do.
12. **Do you love nuts and nut butter? We have good news for you!**
13. **Important information about eye care.**
14. **Alcohol and Diabetes**
15. **Quinoa, a Super Food!**
16. **Problem Solving for Blood Glucose Levels**

◄ July 2013 ►

Sun	Mon	Tue	Wed	Thu	Fri	Sat
	1	2	3	4	5	6
7	8	9	10	11	12	13
14	15	16	17	18	19	20
21	22	23	24	25	26	27
28	29	30	31			

Tuesday, July 02, 2013

Recently Viewed Tools:

- My medication log
- My blood glucose log
- My medication log

Copyright C HY Yu

Appendix A: Representative Screenshots of the Diabetes Online Companion

b) Screenshot of Sample Tracker "Medication Log":

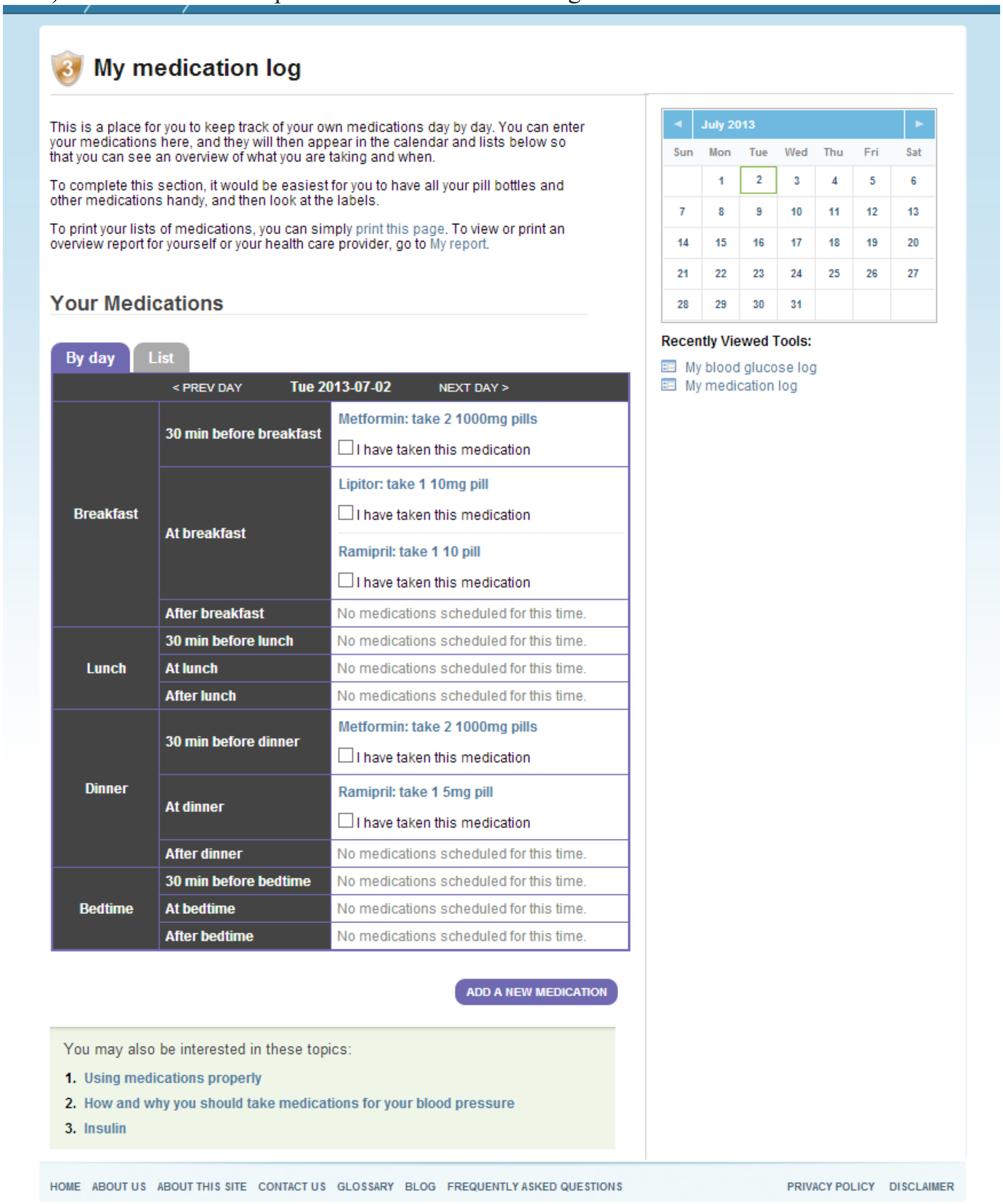

Copyright C HY Yu

Appendix A: Representative Screenshots of the Diabetes Online Companion

c) Sample blog:

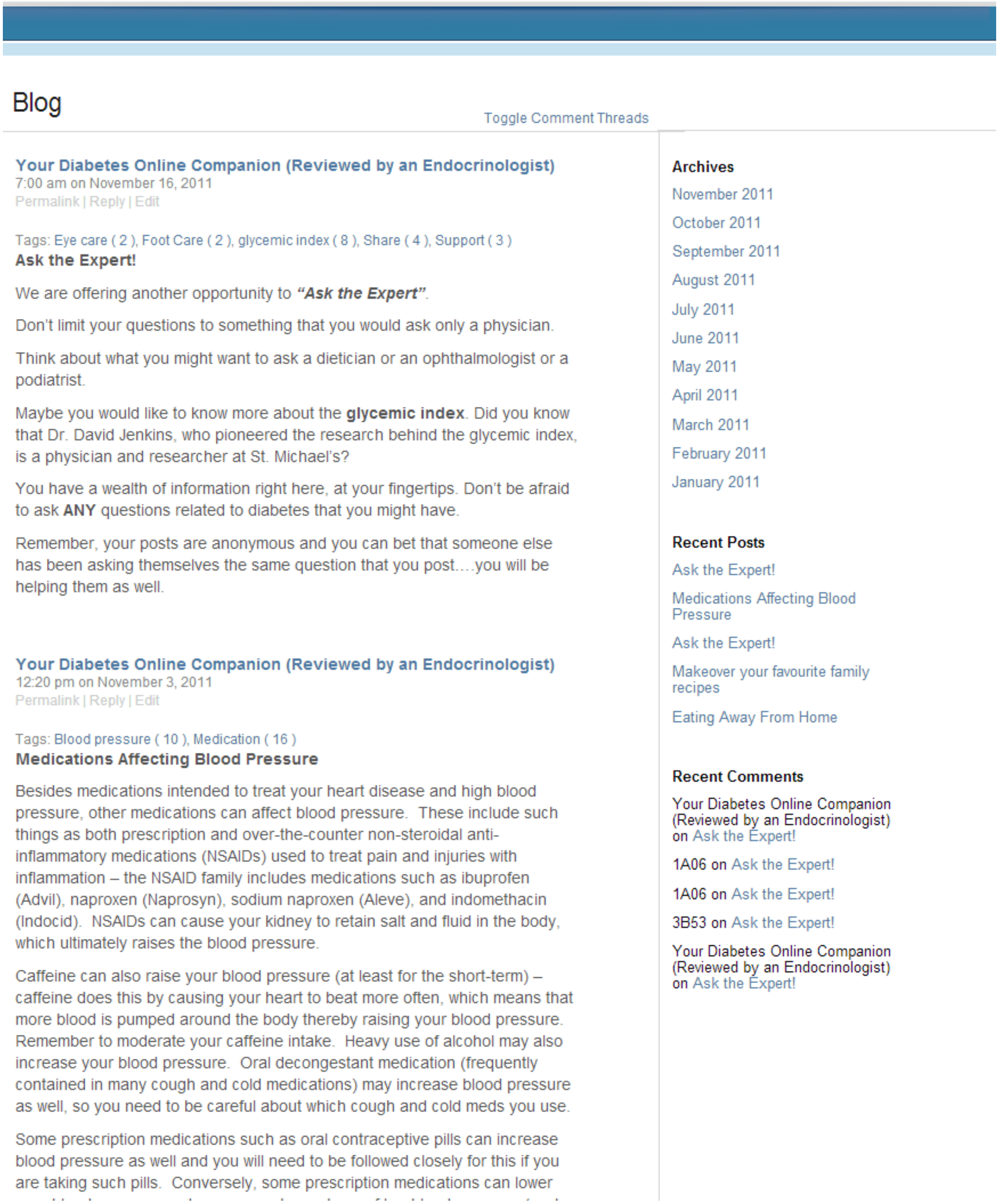

Blog

Toggle Comment Threads

Your Diabetes Online Companion (Reviewed by an Endocrinologist)
7:00 am on November 16, 2011
Permalink | Reply | Edit

Tags: Eye care (2), Foot Care (2), glycemic index (8), Share (4), Support (3)

Ask the Expert!

We are offering another opportunity to ***"Ask the Expert"***.

Don't limit your questions to something that you would ask only a physician.

Think about what you might want to ask a dietician or an ophthalmologist or a podiatrist.

Maybe you would like to know more about the **glycemic index**. Did you know that Dr. David Jenkins, who pioneered the research behind the glycemic index, is a physician and researcher at St. Michael's?

You have a wealth of information right here, at your fingertips. Don't be afraid to ask **ANY** questions related to diabetes that you might have.

Remember, your posts are anonymous and you can bet that someone else has been asking themselves the same question that you post….you will be helping them as well.

Your Diabetes Online Companion (Reviewed by an Endocrinologist)
12:20 pm on November 3, 2011
Permalink | Reply | Edit

Tags: Blood pressure (10), Medication (16)

Medications Affecting Blood Pressure

Besides medications intended to treat your heart disease and high blood pressure, other medications can affect blood pressure. These include such things as both prescription and over-the-counter non-steroidal anti-inflammatory medications (NSAIDs) used to treat pain and injuries with inflammation – the NSAID family includes medications such as ibuprofen (Advil), naproxen (Naprosyn), sodium naproxen (Aleve), and indomethacin (Indocid). NSAIDs can cause your kidney to retain salt and fluid in the body, which ultimately raises the blood pressure.

Caffeine can also raise your blood pressure (at least for the short-term) – caffeine does this by causing your heart to beat more often, which means that more blood is pumped around the body thereby raising your blood pressure. Remember to moderate your caffeine intake. Heavy use of alcohol may also increase your blood pressure. Oral decongestant medication (frequently contained in many cough and cold medications) may increase blood pressure as well, so you need to be careful about which cough and cold meds you use.

Some prescription medications such as oral contraceptive pills can increase blood pressure as well and you will need to be followed closely for this if you are taking such pills. Conversely, some prescription medications can lower [illegible]

Archives

November 2011
October 2011
September 2011
August 2011
July 2011
June 2011
May 2011
April 2011
March 2011
February 2011
January 2011

Recent Posts

Ask the Expert!
Medications Affecting Blood Pressure
Ask the Expert!
Makeover your favourite family recipes
Eating Away From Home

Recent Comments

Your Diabetes Online Companion (Reviewed by an Endocrinologist) on Ask the Expert!
1A06 on Ask the Expert!
1A06 on Ask the Expert!
3B53 on Ask the Expert!
Your Diabetes Online Companion (Reviewed by an Endocrinologist) on Ask the Expert!

Copyright C HY Yu

Appendix A: Representative Screenshots of the Diabetes Online Companion

d) Sample peer story-telling “How do you prevent kidney damage”

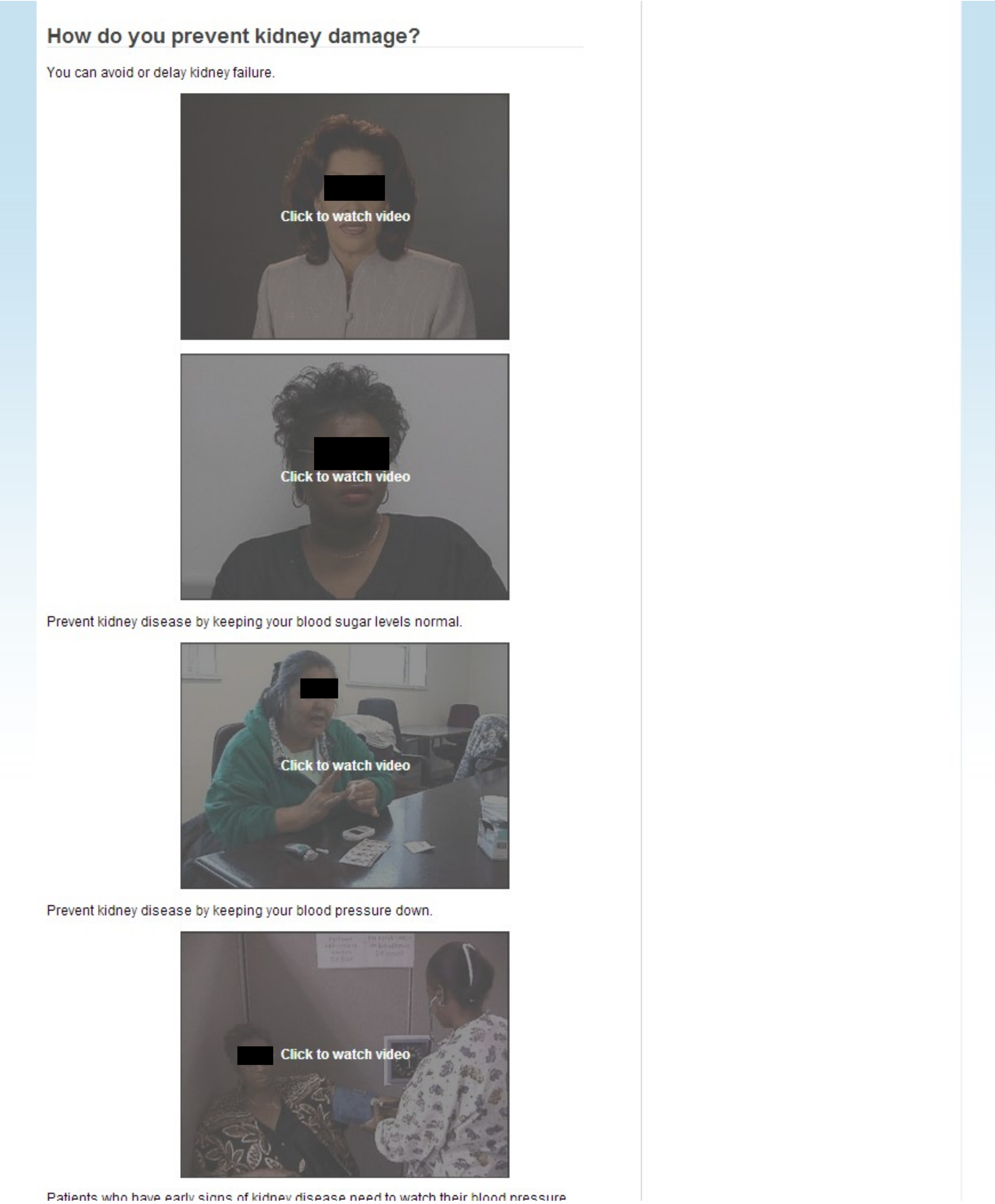

Copyright C HY Yu

Appendix A: Representative Screenshots of the Diabetes Online Companion

e) Sample interactive goal-setting "My profile":

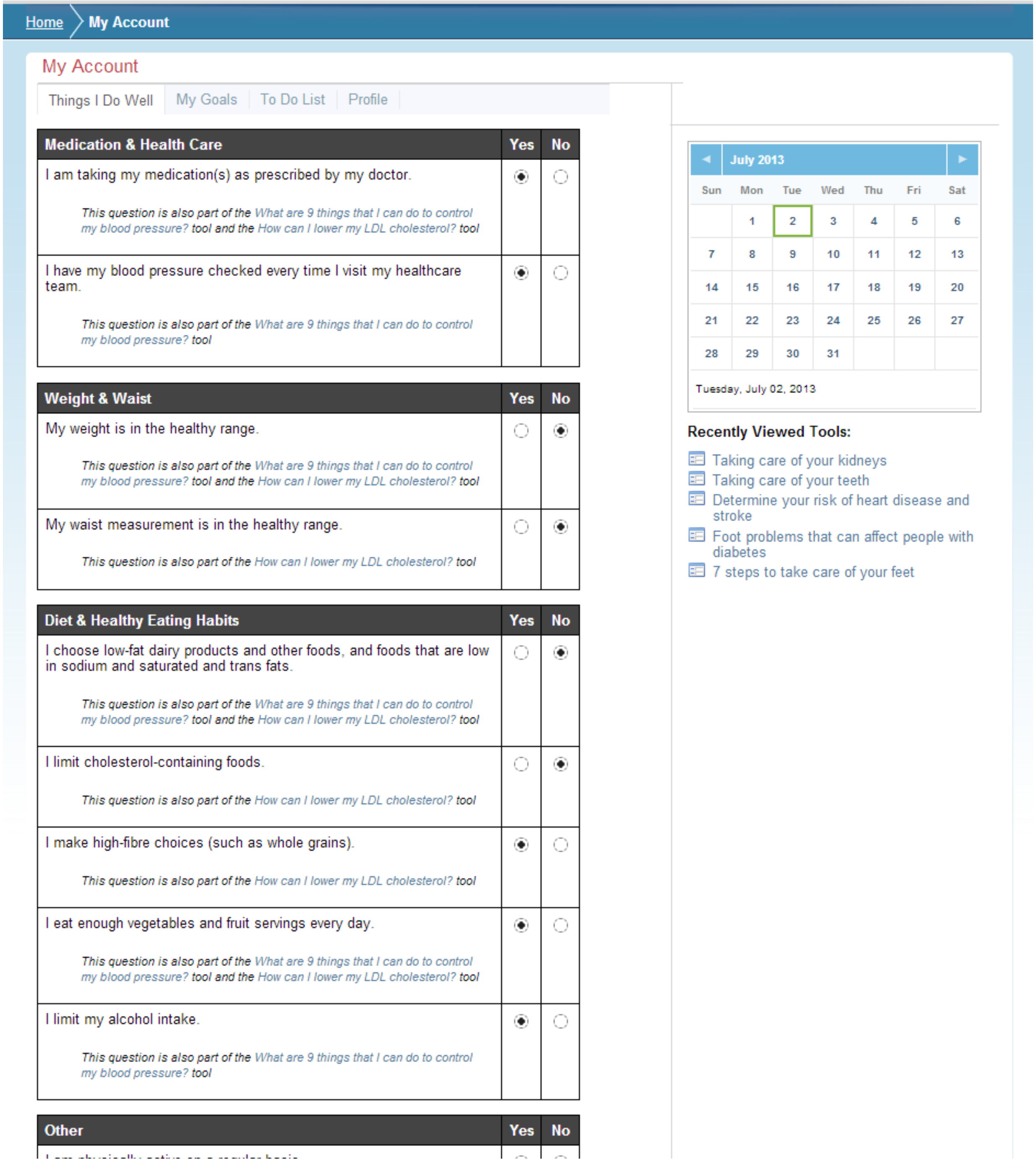

Copyright C HY Yu

Appendix B: Description of primary outcome scales

Outcome	Scale	Description
Self-efficacy	Modified Grossman Self-efficacy for Diabetes Scale(1; 2)	The scale contains 25 items that measure the intensity of self-efficacy for activities of the diabetes regimen. Subjects are asked to describe how much they believe they could or could not do what was stated. The responses on this 6-point scale range from "*very sure I can't*" to "*very sure I can*" do what was stated in each item. Higher scores indicate greater confidence in one's ability to perform the designated treatment activities. The following statements are examples of the self-efficacy items: "Figure meals and snacks at home" and "Keep track of blood sugar levels". The modified self-efficacy scale has a moderate to high reliability (Cronbach's alpha = 0.51 to 0.86).
Self-care behaviour	Summary of Diabetes Self-Care Activities Measure – Revised(3; 4)	Items selected from this self-report instrument assess participants' frequency (over the past 7 days) of engaging in diabetes self-care behaviours, including following a healthy diet, spacing out carbohydrates evenly across the day, physical activity, self-monitoring of blood glucose testing, foot care, and medication and/or insulin taking. For each diabetes self-care behaviour, participants are asked to respond using the following prompt: "*On how many of the last 7 days…*" Responses, which are based on a 7-day week, range from 0 days to 7 days. Greater number of days indicated better self-management. Reliability and validity for this instrument have been found to be adequate, with a test-retest correlation of 0.40 and internal consistency of 0.47.
Diabetes-specific quality of life	Diabetes Distress Scale(5)	The DDS is a 17 item instrument that assesses emotional distress and functioning specific to living with diabetes. Responses are scored on a 6-point Likert-type scale from 1 = "*no problem*" to 6 = "*serious problem*". Scores can range from 17-102 with higher scores indicating poorer diabetes-related quality of life and lower scores indicating better diabetes-related quality of life. The DDS has been found to have high internal reliability with a Cronbach's alpha of 0.93, good convergent validity with the Center for Epidemiological Studies Depression Scale (CESD) (r=0.56) and self-care behaviours including lower adherence to eating recommendations (r=0.30, p<0.001) and lower levels of physical activity (r=0.13, p<0.01)(5). In addition, diabetes distress has been demonstrated to be associated with HbA1c (r=0.17-0.31, *P*=.00-.001), diet (r=-0.38, *P*=.00), physical activity (r=-0.13, *P*=.01) and medication adherence (r=-0.16, *P*=.00)(6; 7)

Appendix C: Semistructured interview guide

Opening questions

As a participant in our study you were invited to visit and use the Diabetes Online Companion to assist you in self-managing your diabetes. We would like to talk to you about how you use the internet to gain knowledge about managing your diabetes.

- First, before entering this study did you ever use the Internet to learn about managing your diabetes? Do you use it for managing any other health conditions you have? Tell me about that.
- How often do you use the internet as a resource to manage your diabetes or other health conditions? Once a month?
- Besides the Internet, what other resources do you use to help you manage your diabetes? *Library, dietician, diabetes educator, classes, friends.*
- Do you have any preferences regarding the types of resources you use (in terms of learning to manage your diabetes)? *Ask only if they say that they use other resources*

Impact on the person with diabetes

We'd like to know if and how the Diabetes Online Companion helped you manage your diabetes or gain knowledge about diabetes.

- **What are your current struggles or concerns with diabetes?**
- **How have you addressed these struggles or concerns?**
- **Did you use the Diabetes Online Companion to help you with these concerns? Why or why not?**
    - What has your experience with the website been, overall? What is your general impression of the diabetes tools?
    - How trustworthy was the content of the website?
    - How does this compare to the way that you feel about other websites?
    -

I have the website open on this computer – please show me which tools you used on the website and tell me what you liked and disliked about each of these these tools.

- "In general, did you find it helpful?
    - Tell me about that. – and then proceed to narrow your probes down after they've had a chance to tell you about it.
        - *If they say it's helpful, ask them how it helped them*
        - *If they say it wasn't, ask them to tell you about why they didn't see it as helpful*
- When did you last use the Diabetes Online Companion?
    - ***If answer is that it has been more than 1 month, could lead to "Barriers and Facilitators of Use" section***
- How did you use the tools on the site?
    - Can trigger thoughts by asking: "which tools did you use", then ask about those specific ones for the questions below.

  - Yes, I think you want to ask them first which ones they used, and then ask about the specific ones and how they used them.
- Please give me an example of how the tools helped you gain knowledge about managing your diabetes.
- Has the website had any effect on how you feel about managing your diabetes? How so? Tell me about that.
  - *For example: Your confidence in taking care of your diabetes, for instance: tracking your blood sugar, taking your medications, checking your feet, monitoring your blood pressure, maintaining your eating plan*

  - **While speaking with other participants, we have been told that a fully realized "companion" website would include both "medical evidence" (the kind of information that a doctor would supply) and "practical advice" (information shared by other people with diabetes)**
    - **Which do you prefer?**
    - **What do you value about each type of information?**
    - **What are the shortcomings of each type of information?**
    - **Which would be more likely to help you and why?**

Behaviour Change

- Did you have a particular self-management goal you were hoping to achieve during the study? Tell me about that.
- Did the website assist you in reaching this goal? How so? Tell me about that
- Show me what tool you used last on the site
- Was this tool helpful? If so, how? If not, how could it be improved?

Interaction with the healthcare system

- Did the Diabetes Online Companion help you with your interactions with your health care professionals? If so, how?
- To clarify for them, can give example: "Some people might feel like it made no difference, that is was just a little extra perk on the side. Some people might have felt that it allowed them to learn more about diabetes on their own, which allowed them to ask their health care provider more specific questions about their own diabetes. Others might have felt that it made their interactions with health care providers less necessary – because their answers were already answered. What do you think?
- Did the website help you prepare for appointments with your doctor or other health care professionals? Tell me about that (which means that they can say it did or it didn't but you can find out why or why not using this probe).
- Did you ever use the website as a bridge to your next appointment
- It's a long time between appointments with your health care providers. If a health issue comes up in between what would you feel comfortable managing yourself? How would you deal with them?
- What would you feel comfortable changing without speaking to your doctor?

Appendix C: Semistructured interview guide

- What types of issues would you go back to your family physician, endocrinologist ie diarrhea, cough?
- Think of an example where you had a question between appts and you went to a website and you got information about your health, what would you do?
- Did you get an opportunity to use the online tools to prepare for your appointments? Ie logs, blog about preparing for appointments?
- Were these tools difficult to find?
- How could we make it easier to find these tools?
- *If participant did not use tools ask* – What do you think of this approach to preparing for your appointments with health care providers?
    - *[if they like this approach]:* why didn't you use this approach?
    - *[if don't like this approach]:* why not?

We designed the website hoping that it would provide better access to timely and personalized health care.

- Did the site help you access care in a more time-efficient manner? Tell me about that.
- Did the site help you to access more 'personalized' care (i.e. care tailored to your own individual needs)? Tell me about that.
- Do you feel that the website enhanced your ability to control your diabetes? Tell me about that.
- In our blog, we introduced the opportunity to "Ask the Expert" any question that you like about diabetes. Was it easy to find this on the blog? Was it readily visible?
    - Did you feel comfortable to ask the expert a question?
    - Did you ask a question of the expert? Tell me about that experience.
    - Can you tell me why you didn't "ask the expert" a question.
    - What topics/things wouldn't you feel comfortable asking in this blog format?

Strengths and Weaknesses of the Website

- We would like to know what you felt were the strengths/weaknesses of the Diabetes Online Companion:
- What content was particularly helpful (or not helpful)? *Refer to the general topics listed on the home page*
- We had general topics listed on the home page of the website ie blood pressure, blood sugar management. What other topics would you like to have seen?
- Sometimes too much information can be overwhelming. What did you find with the Diabetes Online Companion? Was there too much information and you felt overwhelmed or freaked out? Or, was there enough information to be useful to you?
- How do you think this balance of depth of information with not feeling overwhelmed can be best achieved?
- Information on the website was presented in several different formats. Please, tell me how helpful and interesting you found the following formats: Videos, Logs, Blog, Text pages

Appendix C: Semistructured interview guide

Facilitators/Barriers to Use of Website

- How often did you visit the Diabetes Online Companion?
- What kept you returning to the site? Tell me about that.
- What was your motivation for using the site?
- If you visited the site only once, what kept you from visiting it again?
- **Was there anything about the site that kept you from using it?**
- Was there anything about your diabetes that kept you from using the site – ie*denial of diabetes, avoidance of diabetes, poor control*
- What would have enticed you to visit the site more often?
- How would encouragement from your physician or other health care providers to use the site have affected how often you visited?
- **Recognizing that all of us have many demands on our attention and time, what do you feel prevented you from accessing the site more often?** Competing "life" issues? Competing "health" issues? No interest? No time? Not useful? Better sites?
- How would allowing family members or friends to access to the site have an impact on your use of the site?

Privacy/Sharing

We're getting close to the end of the interview now, but I have just a few more questions for you.

  - When you are in a face-to-face situation with whom do you share concerns or thought about diabetes? Family, friends, colleagues, HCP's? Why?
  - What about online situations, for instance a blog or a forum? Would you share information online with people you don't know? Would you want your identity to be protected in this situation?
  - Why do you feel that the 2 situations are different?

- **Blog**
  - Did you know there is a blog on the Diabetes Online Companion?
  - Why didn't you comment, did you feel uncomfortable using the blog?
  - How do you think other people feel about the blog?
  - How do you balance your need for privacy against your need for sharing your feelings or questions about diabetes with others?
  - In focus groups we were told that people with diabetes wanted to share experiences and ideas with one another, yet no one was engaged by the blog. Why do you think this happened?

- **Username/Password**

A username and password were required to enter the Diabetes Online Companion.

  - What did you feel about the username and password system? Was it difficult to use?

- We had the website privacy protected because of privacy issues ie for logs where you were entering personal information, for instance “My blood glucose log”. What solution could you suggest, beyond signing onto the site with a username and password, that would be reasonable and would still protect your privacy?

Sustainability of Use

You have used the Diabetes Online Companion for several months.

- What new content on the website will keep you coming back in the future?
- What did you do when you received an email from the website about new content that was added to the site?
- What would you tell other people with diabetes about the Diabetes Online Companion?

- That’s all of the questions I have for you. But is there anything else that you would like to add before we finish? Thank you for your time.

Appendix D: Effect of Intervention comparing users and non-users (after adjustment for time)

Outcome	Users (n=70)	Non-Users (n=11)	p-value
Self-efficacy	0.146	0.13	0.35
Self-Care	0.18	0.13	0.21
Diabetes Distress	-4.68	-0.87	<0.0001
A1c	0.25	0.21	0.43
Weight	-1.30	0.12	0.17
BP – Systolic	6.69	1.72	0.10
BP – Diastolic	3.27	-1.6	0.014
LDL	0.325	0.121	0.08

Appendix E: References

1. Grossman HY, Brink S, Hauser S: Self-efficacy in adolescent girls and boys with insulin-dependent diabetes mellitus. Diabetes Care 1987;10:324-329
2. Aljasem LI, Peyrot M, Wissow L, Rubin RR: The impact of barriers and self-efficacy on self-care behaviors in type 2 diabetes. Diabetes Educ 2001;27:393-404
3. Toobert DJ, Glasgow RE: Assessing diabetes self-management: the summary of diabetes self-care activities questionnaire. In Handbook of Psychology and Diabetes Research and Practice 1994;
4. Toobert DJ, Hampson SE, Glasgow RE: The summary of diabetes self-care activities measure: results from 7 studies and a revised scale. Diabetes Care 2000;23:943-950
5. Polonsky WH, Fisher L, Earles J, et al: Assessing psychosocial distress in diabetes: development of the Diabetes Distress Scale. Diabetes Care 2005;28:626-631
6. Fisher L, Glasgow RE, Strycker LA: The relationship between diabetes distress and clinical depression with glycemic control among patients with type 2 diabetes. Diabetes Care 2010;3:1034-1036
7. Fisher L, Mullan JT, Arean P, Glasgow RE, Hessler D, Masharani U: Diabetes distress but not clinical depression or depressive symptoms is associated with glycemic control in both cross-sectional and longitudinal analyses Diabetes Care 2010;33:23-28
